# The Ambivalence of Connexin43 Gap Peptides in Cardioprotection of the Isolated Heart against Ischemic Injury

**DOI:** 10.3390/ijms231710197

**Published:** 2022-09-05

**Authors:** Aleksander Tank Falck, Bjarte Aarmo Lund, David Johansen, Trine Lund, Kirsti Ytrehus

**Affiliations:** 1 Cardiovascular Research Group, Department of Medical Biology, UiT The Arctic University of Norway, 9037 Tromsø, Norway; 2 Hylleraas Centre for Quantum Molecular Sciences, Department of Chemistry, Faculty of Science and Technology, UiT The Arctic University of Norway, 9037 Tromsø, Norway; 3 Department of Internal Medicine, University Hospital of North Norway, 9019 Tromsø, Norway

**Keywords:** cardioprotection, connexin43, Gap peptides, ischemic preconditioning, mitochondrial respiration, heart

## Abstract

The present study investigates infarct-reducing effects of blocking ischemia-induced opening of connexin43 hemichannels using peptides Gap19, Gap26 or Gap27. Cardioprotection by ischemic preconditioning (IPC) and Gap peptides was compared, and combined treatment was tested in isolated, perfused male rat hearts using function and infarct size after global ischemia, high-resolution respirometry of isolated mitochondrial and peptide binding kinetics as endpoints. The Gap peptides reduced infarct size significantly when given prior to ischemia plus at reperfusion (Gap19 76.2 ± 2.7, Gap26 72.9 ± 5.8 and Gap27 71.9 ± 5.8% of untreated control infarcts, mean ± SEM). Cardioprotection was lost when Gap26, but not Gap27 or Gap19, was combined with triggering IPC (IPC 73.4 ± 5.5, Gap19-IPC 60.9 ± 5.1, Gap26-IPC 109.6 ± 7.8, Gap27-IPC 56.3 ± 8.0% of untreated control infarct). Binding stability of peptide Gap26 to its specific extracellular loop sequence (EL2) of connexin43 was stronger than Gap27 to its corresponding loop EL1 (dissociation rate constant K_d_ 0.061 ± 0.004 vs. 0.0043 ± 0.0001 s^−1^, mean ± SD). Mitochondria from IPC hearts showed slightly but significantly reduced respiratory control ratio (RCR). In vitro addition of Gap peptides did not significantly alter respiration. If transient hemichannel activity is part of the IPC triggering event, inhibition of IPC triggering stimuli might limit the use of cardioprotective Gap peptides.

## 1. Introduction

Coupled hexamers (connexons) of connexin43 are the dominating constituent of gap junction channels in the healthy heart and are responsible for low resistance electrical coupling and diffusion of low molecular weight substances between neighboring cardiomyocytes. Multiple studies confirm that hexamers of connexin43 are also present as hemichannels in the sarcolemma [1,2]. Hemichannels (non-coupled channels) are normally closed in contrast to open coupled channels in gap junctions. It adds to the complexity that the connexin43 protein has been detected in the subsarcolemmal fraction of mitochondria isolated from hearts. Whether connexin43 in mitochondria forms channels in mitochondria is not fully clarified [3]. The turnover of connexin43 is high [4]. Other cells present in the heart, fibroblasts, endothelial cells and vascular smooth muscle cells are also coupled by connexin43 containing gap junctions [1,5].

Connexin43 is a transmembrane protein with four transmembrane domains, two extracellular loops, one intracellular loop, and intracellular N- and (carboxyl) C-terminal domains. Coupling of the hexamers into gap junctions involves close interaction between the extracellular loops of connexons on neighboring cells. Modification of connexin43 gap junction function, including gating, takes place via post-translational modifications. The long C-terminal loop plays an important role and is functionally connected to the cell signaling network [6]. In addition, the channel-gating control mechanisms appear to depend on whether the connexons are coupled or not. Ischemia induces closure of coupled connexin 43 hexamers of connexin43 in gap junctions leading to a delay in the normal cell-to-cell electrical coupling and eventually isolates the ischemic cell from neighboring cells. However, in contrast to the situation in gap junction, ischemia leads to hemichannel opening [7]. In the open state, hemichannels might release and take up substances below a certain molecular weight and facilitate transmembrane ion transfer along the electrochemical gradient. Channel properties and probability of opening are sensitive to voltage, as have been demonstrated by studies of Ca^++^ influx. Prolonged hemichannel opening will compromise cell integrity and, in combination with an energy deficit, lead to intracellular sodium and calcium overload as part of irreversible cell death.

Using cell cultures of rat heart neonatal myocytes or myofibroblasts, we have previously demonstrated that reversible gap junction uncoupling occurs with hypoxia or ischemia and that uncoupling expands with prolonged hypoxia, but also that uncoupling can be delayed in cells pretreated with preconditioning-like protocol involving transient hypoxia [5,7]. Ischemic preconditioning delays ischemic injury and protects against reperfusion injury [8,9]. Gap junction closure, hemichannel opening, and, lastly, cell death can be observed as time-dependent sequential events with exposure of cell cultures to ischemia-like culture conditions [7].

Peptides, called Gap peptides or connexin mimetic peptides, interfere with the function of a connexin or the coupling between two hexamers belonging to two different cells. Gap19 (9 amino acids), Gap26 (13 amino acids) and Gap27 (11 amino acids) are analogues to specific domains in connexin43, the intracellular N-terminal tail, the first extracellular loop (EL1) and the second extracellular loop (EL2), respectively. The three peptides are expected to interfere with channel function and docking by substituting for bounds between different parts of the connexin molecule. All three peptides have been reported to limit ischemic injury when given acutely [2,7,10,11].

In the present study, we tested Gap peptides 19, 26 and 27 and compared the infarct sparing effect of cardioprotection by sublethal stress stimulus ischemic preconditioning (IPC) using the isolated perfused rat heart as an experimental model. To test if channel activity might take place during ischemic preconditioning, we combined the two treatments, the connexin43 hemichannel inhibiting Gap peptides plus ischemic preconditioning. In line with the central role of mitochondria in ischemia-reperfusion injury, we also tested if respiration of subsarcolemmal mitochondria isolated from perfused hearts responded to ischemic preconditioning and if in vitro presence of Gap peptides changed mitochondrial respiration. The binding strength of the peptides Gap26 and Gap27 to the respective specific sequences of the extracellular loops of connexin43 was examined to elucidate further the role of connexin43 and/or hemichannel opening in both the triggering preconditioning and promoting ischemic injury. The results indicated that Gap26 interfered with the triggering phase of preconditioning and thereby blocked cardioprotection.

## 2. Results

**Figure 1:**
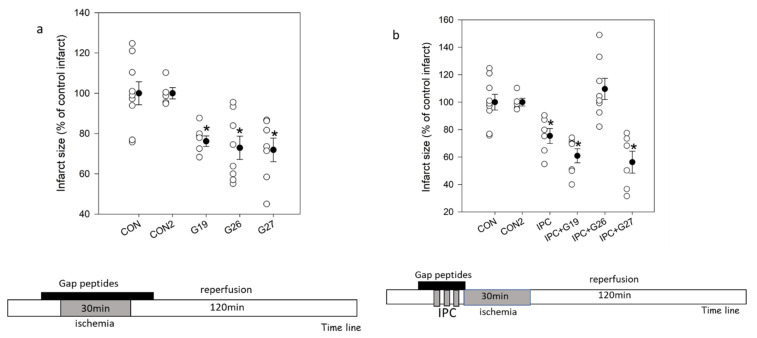
Infarct size in % of untreated control infarcts. Isolated buffer perfused hearts were subjected to global ischemia followed by reperfusion. Infarct size relative to the total ventricular volume was measured and results expressed in % of untreated control infarcts. (**a**) Controls subjected to ischemia-reperfusion with no treatment and hearts treated with Gap19, Gap 26 or Gap 27 (0.05 µM) before ischemia initially at reperfusion. (**b**) Hearts subjected to ischemic preconditioning IPC prior to 30 min ischemia-reperfusion with no treatment or with Gap19, Gap 26 or Gap 27 (0.05 µM) added to the buffer only during the PC treatment. Open symbols represent individual hearts, closed symbols are group mean ± SEM, * *p* < 0.05 vs. control.

*Infarct size:* Results are presented in Figure 1a,b. The three Gap peptides 19, 26 and 27 reduced infarct size significantly to 76.2 ± 2.7, 72.9 ± 5.8 and 71.9 ± 5.8% of control infarcts, respectively, and to the same degree as IPC (75.4 ± 5.5%). When given as a pretreatment in combination with IPC, infarct size reduction was present with the use of Gap19 (60.9 ± 5.1%) and Gap27 (56.3 ± 7.8%). In contrast, when Gap26 was added in conjunction with IPC, protection was lost (109.6 ± 8.0%).

**Figure 2:**
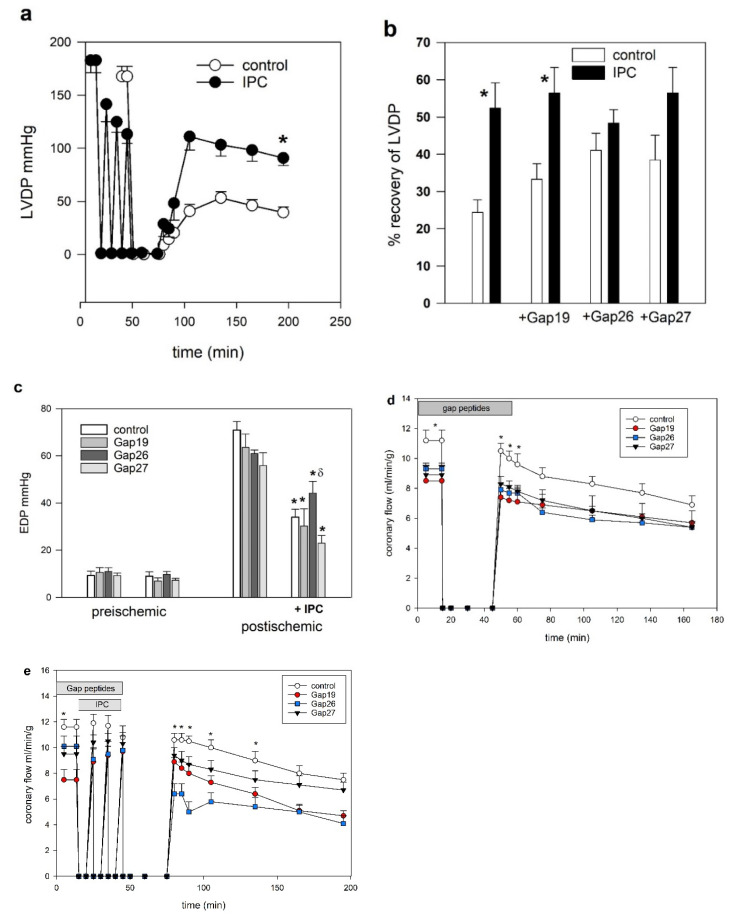
(**a**–**c**) Heart function in isolated hearts subjected to global ischemia followed by reperfusion with or without IPC and/or Gap peptide treatment: (**a**) Left ventricle developed pressure (LVDP) in control hearts (closed symbols) and hearts subjected to ischemic preconditioning (IPC) (open symbols) followed by 30 min ischemia and 120 min reperfusion * *p* < 0.05 vs. control. (**b**) Postischemic recovery (after 30 min ischemia and 120 min reperfusion) of LVDP as % initial preischemic values. Untreated hearts (controls) and hearts treated with Gap peptides 0.5 uM before and after 30 min ischemia are shown as open bars. Ischemic preconditioning (IPC) treated hearts are shown with closed bars. * *p* < 0.05 comparing IPC with hearts without IPC treatment. (**c**) End diastolic pressure (EDP, mmHg) prior to ischemia and at the end of 120 min reperfusion in the eight test groups. Endpoint contracture was significantly reduced by IPC in all groups. * *p* < 0.05 compared with postischemic hearts not treated with IPC. End diastolic pressure was significantly higher in IPC Gap26 treated hearts compared to IPC Gap27 treated hearts δ *p* < 0.05. (**d**,**e**) Coronary flow: (**d**) with/without Gap peptides given prior to 30 min ischemia and initially during reperfusion. * *p* < 0.05 by ANOVA comparing groups at the same timepoint, Gap19 vs. no Gap peptide prior to ischemia and Gap peptides vs. control at reperfusion. (**e**) Coronary flow with/without Gap peptides given during triggering IPC. * *p* < 0.05 by ANOVA, Gap19 vs. no Gap peptide prior to IPC, Gap26 vs. no Gap peptide at reperfusion).

*Heart function*: Functional parameters are presented in Figure 2a–e. With respect to recovery of contractile function (LVDP and EDP), no differences were observed between the non-IPC Gap-peptide treatment groups, whereas IPC treatment and IPC combined with Gap19 resulted in a significant increase in % recovery of LVDP (*p* < 0.05). Post ischemic contracture evaluated by end-diastolic pressure (EDP) at endpoint was significantly lower in hearts treated with IPC; however, there was also a significant difference between IPC plus Gap26 and Gap27, indicating again that Gap26 interfered with IPC. Compared to untreated hearts, the addition of Gap peptides tended to lower coronary flow at baseline. Coronary flow at baseline was significantly reduced with Gap19 compared to control (*p* < 0.05). At reperfusion of non-IPC hearts, Gap peptide-treated groups demonstrated reduced coronary flow during the initial 10 min. Coronary flow in hearts treated with IPC plus Gap26 was significantly reduced compared to control without peptides during the first 60 min of reperfusion.

**Figure 3:**
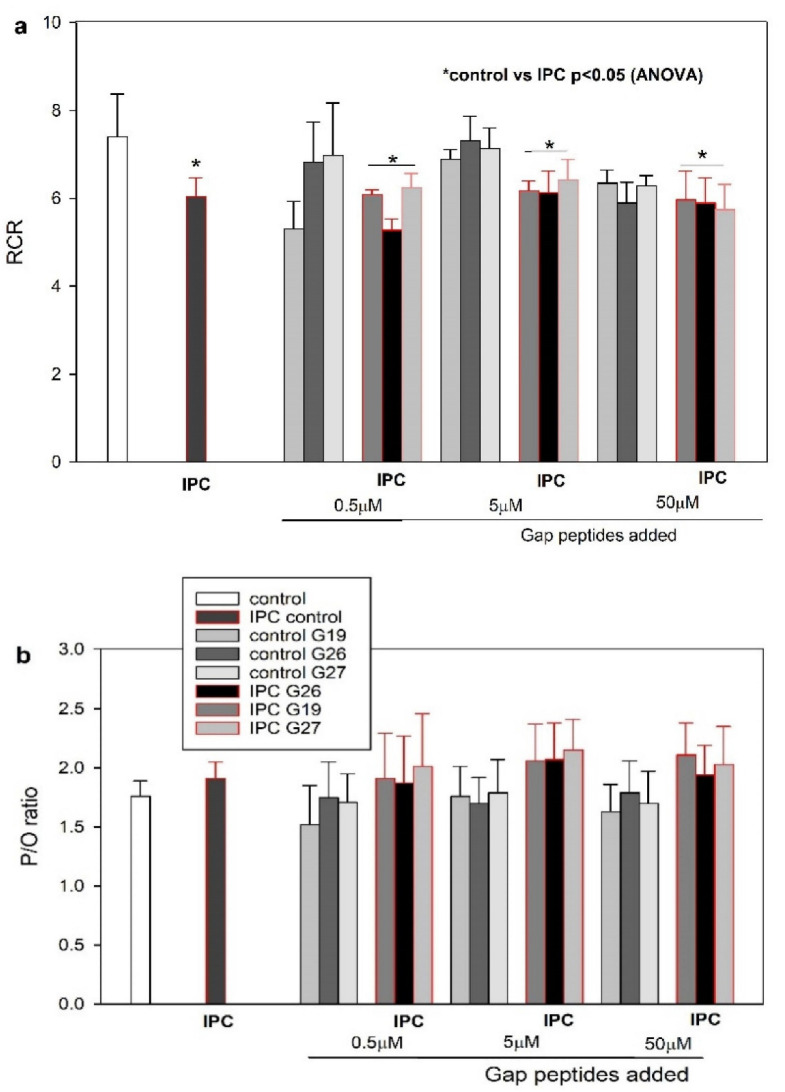
Subsarcolemmal mitochondrial respiratory control ration (RCR) and phosphate/oxygen (P/O) ratio. (**a**) Respiratory control ratio (RCR) as max O_2_ flux (OXPHOS) at saturating [ADP] divided by state 2 leak respiration (glutamate—maleate as substrates). * *p* < 0.05 by ANOVA compared to mitochondria from hearts not subjected to IPC. (**b**) Phosphate/oxygen (P/O) ratio as µmol ADP added divided by µmol oxygen used (in the presence of glutamate—maleate). The grouped bars represent the presence of increasing concentrations of Gap peptides (0 and 0.5, 5, 50 µM as indicated) in the respiration chambers: Gap19 (G19), Gap26 (G26), Gap27 (G27).

*Mitochondrial high-resolution respirometry*: Results are presented in Figure 3a,b and Table 1. Untreated perfused control hearts and hearts subjected to the IPC procedure were used for isolation of subsarcolemmal mitochondria and respirometry were performed with or without the gap peptides added. Measurements of average oxygen flux (nmol O_2_ min^−1^ normalized to CS activity µmol IUmin^−1^) in the 20 test groups (*n* = 3) are presented in Table 1. These measurements represent background data for the calculation of RCR and P/O ratio. Respiration in subsarcolemmal mitochondria was significantly affected by the preconditioning protocol in that RCR (3a) was reduced from 7.1 ± 0.28 to 6.2 ± 0.21 (*p* = 0.01). P/O ratio (3b) was 1.71 ± in mitochondria from control hearts and 2.0 ± 0.16 in mitochondria isolated from IPC hearts (*p* = 0.17). Gap peptides did not significantly affect average oxygen flux, RCR or P/O ratio when added in vitro to the buffer of isolated mitochondria from control hearts or from IPC hearts (Figure 3a,b, Table 1).

**Figure 4:**
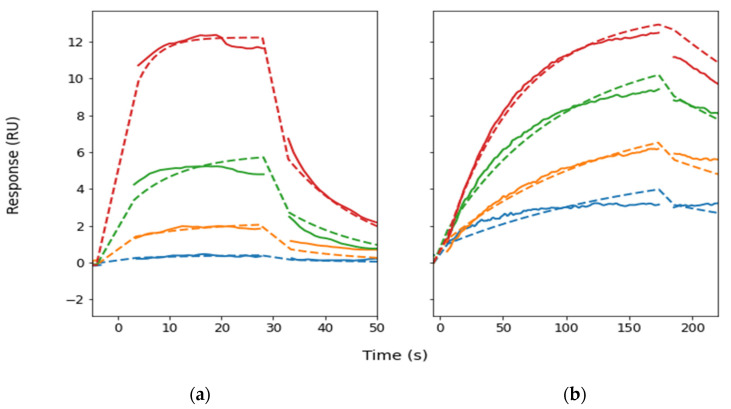
Biophysical characterization of Gap26 and Gap27 peptide binding. Gap peptides were injected over NTA surfaces with captured EL1 (**a**) and EL2 (**b**) on RadA-scaffolds. (**a**) RadA-EL1 with Gap27 (0.16–20 µM) and (**b**) RadA-EL2 with Gap26 (3–50 µM) showed clear differences in off-rates. The fit to a 1:1 binding model are shown as dashed lines. Colors indicate the concentrations of the injections in the order, from lowest concentration to highest: blue–orange–green–red.

*Biophysical characterization of Gap peptide* binding: Results are presented in Figure 4 and Table 2. Constructs of the monomeric ATPase domain of *Pyrococcus furiosus* RadA containing either EL1 and EL2 were expressed recombinantly in milligram amounts from *E. coli* and were purified to homogeneity as described previously (Rossmann et al. 2017). Binding curves from surface plasmon resonance experiments with the Gap peptides Gap26 and Gap27 are shown in Figure 4. The curves could be modeled with a simple 1:1 binding model, and the fitted kinetic parameters are shown in Table 1. Increasing the concentration of the Gap27 peptide further led to curves symptomatic of non-specific binding (data not shown). A key finding is the order of magnitude difference in off-rates between EL1-Gap27 and EL2-Gap26, even in the presence of a reducing agent (0.1 mM TCEP) for EL2-Gap26. Binding curves (Figure 3) show more prolonged binding of the Gap26 peptide to its complementary binding region in the surrogate compared to Gap27 and clearly illustrate a difference in function of these two peptides.

## 3. Discussion

The current study confirms that Gap peptides Gap19, Gap26 and Gap27 are cardioprotective peptides reducing infarct size to the same degree as ischemic preconditioning in an isolated perfused rat heart [2,7,10,11,12]. The Gap peptides were given immediately prior to introducing ischemia and during the first 10 min of reperfusion to secure their presence both during ischemia and at reperfusion. The proposed mechanism of action of the peptides is to limit ischemia-induced hemichannel opening [10,13,14]. Connexin43 hemichannels are reported to open in response to an increase in intracellular Ca^++^, reduction in extracellular Ca^++^, membrane depolarization, reduction in redox potential and metabolic inhibition [14]. The suggested mechanism of the peptides is that binding to the specific binding sites contributes to maintaining hemichannels in the closed state and increases the threshold for ischemia induces opening. The present results are in line with such a mechanism of action.

In the present study, the coronary flow was reduced in the buffer-perfused hearts when Gap peptides were present, but contractile function was not affected. Stress-induced release of ATP and prostaglandin as part of vasomotor regulation occurs through hemichannel opening. Indeed, it has repeatedly been observed that Gap peptides are able to modify vascular function [15,16,17,18]. The isolated buffer-perfused heart depends on a dilated vasculature and high coronary flow for oxygen delivery by dissolved oxygen only, and the implication of the observed reduction in coronary flow with the Gap peptides in this study is uncertain.

IPC is a powerful and robust cardioprotective mechanism. In the current study, we wanted to examine if IPC in combination with Gap peptides would interfere with infarct size limitation. This turned out to be the case, and the results revealed a difference between the peptides with respect to infarct size limitation, which was not present when used without IPC. Peptides were added prior to and during three cycles of ischemic preconditioning pretreatment before prolonged global ischemia but not at the following reperfusion. When combining Gap26 and ischemic preconditioning, the protection against infarction disappeared, whereas protection was maintained with Gap19 and Gap27. Since both Gap26 and Gap27 bound to extracellular loops of connexin43 molecules, we then tested the two peptides’ binding stability for respective loops to try to explain why they behaved differently in combination with an IPC protocol. Biophysical characterization of the binding of Gap26 and Gap27 to amino acid sequences analog to their corresponding extracellular loops confirmed a difference between the two peptides. Gap26 binds with significantly lower kinetic rates than Gap27, even in the presence of a reducing agent, thus forming a more stable binding. The results might indicate that short-lasting reversible connexin43 hemichannel opening could contribute to triggering IPC. However, the mechanism behind the blocked preconditioning effect by Gap26 could also be unrelated to peptides sequence-specific connexin43 binding and due to binding to or steric inhibition of membrane receptors or channels of importance in triggering ischemic preconditioning. Gap26 contains cysteine, and in an oxidizing environment, it appears likely that disulfide bridges may form. Antioxidants block the cardio-protective triggering stimuli [19,20]. We have previously shown that antioxidant uric acid blocks cardioprotection by preconditioning but that adding the unspecific anion-channel blocker probenecid could inhibit this effect [21]. Depending on the species, antagonists to specific cell-membrane receptors also block the triggering of ischemic preconditioning, indicating the presence of an autocrine mechanism. With structural similarities between channel-forming proteins, we cannot fully exclude that the effects we observed were related to, for example, the pannexin1/P2X7 receptor complex [22,23,24,25]. A limitation in the present study is that we have not used scrambled peptides in our study to look for unspecific effects, but instead three different peptides, of which only one blocked IPC.

The detailed mechanisms behind IPC are still debated, but multiple studies confirm that mitochondria [26] play a crucial role in the observed infarct size limitation [26,27,28]. IPC promotes translocation of signaling kinases to mitochondria, changes volume regulation of mitochondria, opens mitochondrial potassium channels [29] and delays ROS-induced mitochondrial permeability transition [30]. Connexin43 can be detected in subsarcolemmal mitochondria using immunoblotting of extracts from isolated mitochondria [31]. The role of connexin43 in mitochondria has recently been reviewed [3]. It has been proposed that IPC depends on the presence of mitochondrial connexin43. Our results partly support this. Interaction between connexin43 and Gap peptides during IPC could take place in subsarcolemmal mitochondria in addition to the cell membrane. Boengler and coworkers, among others, proposed that Cx43 forms hemichannels in the inner mitochondrial membrane and that these channels take part in the cardioprotective mechanism of IPC, [32]. Cx43 transgenic (+/−) mice lost the effect of IPC [33]. A protective role of mitochondrial Cx43 in IPC, which is antagonistic to the role of sarcolemmal Cx43- hemichannels in ischemia, was thus proposed. Fibroblast growth factor 2 triggers the protection of rat heart mitochondria, and this protection has been shown to be connexin-43 dependent [34]. Fibroblast growth factor 2 induced protection against calcium-induced mitochondrial permeability transition was reported inhibited by Gap27 in isolated subsarcolemmal mitochondria [34], but substantially higher concentrations of Gap27 were needed compared to what was used in the present study. Gap26 binds more strongly, and this peptide blocks protection in the present study.

In the present paper, we used isolated subsarcolemmal mitochondria, and we tested if ischemic preconditioning prior to mitochondrial isolation and later presence of Gap peptides influenced respiration. We detected a slight but significant reduction in mitochondrial respiratory control ratio and a tendency toward increased P/O ratio with ischemic preconditioning, but there was no significant direct influence of the three peptides, and the effect of IPC tended to be maintained. Our results partly agree with Liem et al. (2008) [28], proposing that mild uncoupling might take place with IPC and with other studies of mitochondrial function after IPC [35]. Thus, although connexin43 might have a role in IPC modulation of respiration in subsarcolemmal mitochondria as previously suggested [33,36], we were not able to detect significant change using Gap peptides (gap19, gap26, gap27) concentrations 0.5, 5 and 50µM. Other relevant mitochondrial effects, especially permeability transition or swelling, were not tested in the present study.

We tested the cardioprotective potential of the peptides in healthy male rat hearts. In the current study, possible sex and species differences in response were not examined. Connexin43 is reported to be expressed at higher levels in female mice and rats compared to males [37,38,39]. The expression of the connexins changes over time due to rapid turnover, and in various disease states, the expression pattern of connexins is changed. Cx43 is the dominating connexin expressed in cardiomyocytes, but other connexin subtypes, pannexin, and channel-forming proteins are also present in the heart and might also undergo ischemia-related changes.

Through the years, multiple attempts have been made to use cardioprotection by IPC or pharmacological interventions that mimic IPC to reduce infarct size in the clinic [40]. So far, this has been without success when carried through to large phase 3 clinical studies. Adaption to stress is a beneficial endogenous mechanism closely related to injury mechanisms. Several common compounds inhibit the triggering step of IPC, although, by themselves, they are cardioprotective or without known direct effects on the heart muscle, for example, melatonin, acetylsalicylic acid (aspirin) and soluble uric acid [21,40,41,42,43]. In this respect, the gap peptides add to the list of compounds with dual roles in cardioprotection.

In conclusion, Cx43 Gap peptides are powerful cardioprotective agents, but more knowledge about adaptation to stress stimuli in the heart is needed to develop fully the potential of Gap peptides as cardioprotective agents.

## 4. Materials and Methods

### 4.1. Animals

All experiments were approved by the Norwegian animal research authority and were performed according to the Norwegian Animal Welfare Act and the European Convention for the Protection of Vertebrate Animals Used for Experimental and other Scientific Purposes (ETS No. 123 and 2010/63/EU). Male Wistar rats 250–350 g (Charles River, UK) were anesthetized with pentobarbital (100 mg/kg i.p.) and heparinized (300 IU i.p.), the chest opened and the heart removed.

### 4.2. Langendorff Perfusions

The heart was immediately submerged in an ice-cold buffer and quickly mounted on the perfusion apparatus, where it was perfused with the Krebs–Henseleit buffer (gassed with 5% CO_2_ and 95%O_2_) in a constant pressure Langendorff setup (100 cm H_2_O). The temperature was kept at 37 °C during the whole experiment by keeping the heart submerged in a heated buffer. Left ventricular pressure was recorded using a balloon catheter connected to a pressure transducer. Coronary flow was measured by timed collections of perfusate.

Hearts were stabilized for 15 min, then either subjected to 30 min of global ischemia and 2 h of reperfusion or preconditioning with 3 cycles of 5 min of ischemia and reperfusion (IPC) before 30 min of global ischemia and 2 h of reperfusion. Care was taken to secure controls corresponding in time and rat batch with the intervention experiments.

### 4.3. Peptides

Connexin mimetic peptides Gap19 (amino acid sequence KQIEKKFK), Gap26 (VCYDKSFPISHVR) and Gap27 (SRPTEKTIFII) from Pepnome Inc., Hong Kong, China, were dissolved in the Krebs–Henseleit buffer to a final concentration of 0.5 µM [7,12] and used in experiments the same day. Amino-acid sequences were confirmed by mass spectrometry analysis. The hearts that were subject to ischemia without IPC received the peptides prior to and after 30 min global ischemia. The hearts that were subject to IPC received the peptides during the preconditioning cycles only prior to 30 min ischemia.

### 4.4. Infarct Size Measurements

At the end of reperfusion, the heart was immediately cut off caudal to the atria yielding only ventricular tissue. The ventricular tissue was then weighed in its wet state and immediately frozen to −18 °C for 24 h, sliced into four or five 2 mm thick slices and dyed with 1% 2, 3, 5 triphenyl tetrazolium chloride (TTC) (Sigma-Aldrich Co., Ltd., St. Louis, MI, USA) in Dulbecco’s phosphate buffered saline (PBS) (Sigma-Aldrich Co.). Each heart was submerged in 50mL of 1% TTC in PBS for 20 min at 37 °C in a light-free environment. Thereafter the slices were quickly dried and fixed in 4% formaldehyde for 30 min before they were scanned by a high-resolution scanner; the images were then blind-coded. Infarcts were manually traced, and the area of infarct relative to the whole area was quantified using Image J software.

### 4.5. Mitochondrial Isolation and Respiration

Hearts were control perfused or subjected to IPC. Isolation of mitochondria was performed using the procedure described by Palmer et al. (1977) [44] with minor modifications. All procedures were performed on ice or at 4 °C. Briefly, the ventricles were removed, weighed and placed in the buffer containing (in mM): 100 KCl, 50 MOPS, 1 EGTA, 5 MgSO_4_, 1 ATP and pH 7.4. Heart tissue was minced with scissors in a small volume of buffer. Buffer plus bovine serum albumin BSA (2 mg/mL) were added, and the tissue suspension was homogenized with Polytron for 7 s and thereafter with two strokes of a Potter-Elvehjem homogenizer (Glas-Col, Terre Haute, IN, USA) and centrifuged at 590× *g* for 10 min at 4 °C. The supernatant was filtered through a nylon net (NITEX 300 lm; Yulee, FL, USA). Supernatants now containing mitochondria mainly from the subsarcolemmal compartment (SSM fraction) were centrifuged at 3000× *g* for 10 min at 4 °C, and the SSM pellet was again resuspended in the buffer plus BSA and centrifuged at 3000× *g* for 10 min. The pellet was resuspended in the buffer containing (in mM): 100 KCl, 50 MOPS 50, 0.5 EGTA and pH 7.4 and centrifuged at 3000× *g* for 10 min at 4 °C. The final pellet was resuspended in this buffer and left on ice for 30 min for stabilization of membranes.

Measurement of mitochondrial oxygen consumption was performed in an oxygraph (Oxygraph 2k; Oroboros Instruments, Innsbruck, Austria) using a respiration medium [100 KCl; 50 MOPS; 1 EGTA; 5 KH_2_PO_4_ (all in mM); BSA 1 mg mL) and pH = 7.4] in a closed 2 mL chamber at 37 °C. Glutamate (5.0 mM) and malate (2.5 mM) were used as substrates. After obtaining stable respiration O_2_ flux (nmol O_2_/min/mL), ADP was added to a final concentration of 50 µM to measure coupled respiration. After depletion of ADP and stable respiration, a saturating ADP amount (2.5 mM) was added to obtain max coupled respiration (OxPhos). Finally, oligomycin was added to a final concentration of 4 µg/mL to inhibit the ATP-synthase, and a stable state oligomycin measurement was recorded. At the end of the experiments, protein content was estimated by the Bradford method [45], and citrate synthase activity was measured [46]. Results of O_2_ flux measurements (nmol O_2_/min) were normalized to citrate synthase activity (IU µmol/min) in the sample. Phosphate/oxygen ratio (P/O ratio) was calculated as ADP phosphorylated divided by oxygen molecules consumed. The respiratory control ratio (RCR) was calculated as the ratio between max state 3 (OxPhos) and uncoupled respiration.

In order to assess the effect of Gap26 and Gap27 on mitochondrial respiration, peptides in concentrations 0.5, 5.0 and 50.0 µM were added to the isolated mitochondria. After 10 min preincubation on ice 60 µL of the suspension was added to the oxygraphy, and peptide concentration was adjusted to the new volume, so the final peptide concentration was the same during the whole experiment. Each subgroup consisted of samples from 3–4 hearts, and care was taken to minimize variability by testing the different Gap peptides and concentrations concomitantly in every heart at the same time using multiple respiratory chambers.

### 4.6. Binding Kinetics

For the binding kinetics, a surrogate approach was used. The extracellular loops (EL1: LGTAVESAWGDEQSAFRCNTQQPGCENVCYDKSFPISHVR, EL2: GFSLSAVYTCKRDPCPHQVDCFLSRPTEKTI) of connexin43 were grafted into the scaffold of the monomeric ATPase domain of *Pyrococcus furiosus* RadA (Rossmann et al. 2017) by gene synthesis (GenScript) and inserted into a pET-24(+) vector for expression in *Eschericia coli* NiCo21(DE3) (New England BioLabs). Cells were grown in shaking incubators in 2 × 500 mL ZYP-5052 media for 4 h at 37 °C before the temperature was reduced to 17 °C for overnight expression. Cells were harvested by centrifugation at 7500× *g* for 45 min at 4 °C. Pellets were resuspended in 50 mM HEPES pH 7.5 supplemented with 500 mM NaCl (buffer A), and sonicated for 15 min at a maximum of 20 °C. The cell lysates were heat treated for 10 min at 60 °C before clarification by centrifugation at 50,000× *g* for 45 min at 4 °C and filtration through 0.45 µm syringe filters. The clarified lysate was loaded on HisTrap FF 1 mL crude columns on an äkta Pure system equilibrated with the buffer A. Protein was eluted by an increase in imidazole concentration from 50 mM to 375 mM. Protein homogeneity was confirmed by SDS-PAGE. Characterization of binding to GAP peptides was performed using surface plasmon resonance with the Biacore T200 (Cytvia) using NTA-capture of the his-tagged RadA-connexin43 constructs. The running buffer was 50 mM HEPES pH 7.5 with 500 mM NaCl and 0.005% Tween-20; for the interaction of EL2-Gap26, 0.1 mM TCEP was included to prevent oxidation of the cysteines. The RadA-connexin43 proteins were used as the ligand and were captured to a level of approximately 200 RU. For EL2-Gap26, the temperature was set to 30 °C to increase kinetic rates, while for EL1-Gap27, it was kept at 25 °C. In all cases, no nickel was injected over the first flow channel, and this was used as a reference channel. Flow rates of 30 µL/min were used for the injection of Gap peptides as the analyte. For EL1-Gap27, concentrations between 3–50 µM were used, and for EL2-Gap26, concentrations between 0.16–20 µM were used to minimize the contribution of non-specific binding.

### 4.7. Statistics

Statistical analysis was performed by SigmaPlot version 14.5 (Systate Software Inc. Chicago, IL, USA) using one-way or two-way ANOVA followed by the Holms Sidac method as a post hoc test when appropriate. Results are presented as mean ± SEM except for binding kinetics, which is presented as mean ± SD (*n* = 3–4).

## Figures and Tables

**Table 1:** Oxygen flux (nmol O^2^ min^−1^ normalized to CS activity µmol IU min^−1^).

Oxygen Flux [nmol O_2_ (µmol IU CS Activity)^−1^]	Glutamate + Maleate		+ADP 50 µM		State 4 (LEAK _ATP_)		OXPHOS Saturating +ADP 2.5 mM		+Oligomycin	
	crt	IPC	crt	IPC	crt	IPC	crt	IPC	crt	IPC
Without peptide										
	16.1 ± 2.9	17.4 ± 3.4	69.9 ± 16.0	75.1 ± 15.8	20.8 ± 3.6	22.7 ± 2.5	114.5 ± 22.3	106.6 ± 18.6	42.5 ± 5.0	39.0 ± 6.3
Gap19 (µM)										
0.5	16.6 ± 5.0	15.2 ± 4.2	68.9 ± 6.5	69.8 ± 28.0	23.8 ± 5.2	21.2 ± 5.3	89.2 ± 35.6	92.5 ± 25.1	42.8 ± 4.5	37.8 ± 7.4
5	16.6 ± 2.6	15.9 ± 1.7	75.1 ± 11.3	71.4 ± 7.5	22.1 ± 1.6	21.0 ± 2.6	113.7 ± 12.9	98.3 ± 10.5	43.5 ± 2.6	39.0 ± 3.7
50	18.2 ± 0.9	19.6 ± 6.1	78.9 ± 14.0	76.8 ± 21.6	24.8 ± 3.0	24.0 ± 6.1	115.3 ± 8.7	116.2 ± 37.7	44.6 ± 5.1	44.2 ± 7.5
Gap26 (µM)										
0.5	15.0 ± 3.2	17.7 ± 4.5	63.7 ± 6.7	71.6 ± 28.5	21.4 ± 1.0	22.8 ± 4.4	99.2 ± 18.0	93.0 ± 20.7	41.3 ± 5.1	37.2 ± 4.7
5	16.3 ± 3.5	16.3 ± 2.6	77.1 ± 10.6	71.3 ± 7.5	21.2 ± 1.7	21.3 ± 2.8	118.6 ± 25.5	98.2 ± 6.1	45.0 ± 7.5	38.0 ± 6.0
50	16.8 ± 1.9	18.8 ± 4.1	74.5 ± 10.2	80.8 ± 19.9	21.6 ± 0.9	24.1 ± 3.5	100.0 ± 13.1	109.5 ± 23.1	37.1 ± 10.1	41.1 ± 11.4
Gap27 (µM)										
0.5	16.3 ± 6.2	15.1 ± 2.1	67.7 ± 11.3	66.2 ± 13.2	22.7 ± 4.6	19.3 ± 1.8	103.3 ± 20.4	93.6 ± 4.1	43.1 ± 4.1	35.0 ± 6.7
5	15.8 ± 3.2	15.5 ± 3.7	68.8 ± 10.3	71.0 ± 15.3	20.8 ± 1.1	20.1 ± 2.5	112.9 ± 28.2	96.7 ± 11.0	41.8 ± 5.8	38.8 ± 8.0
50	14.7 ± 1.5	17.0 ± 15.2	46.4 ± 12.1	68.9 ± 17.9	21.3 ± 2.8	22.5 ± 3.6	92.1 ± 12.0	94.8 ± 22.7	41.6 ± 5.1	40.8 ± 7.6

**Table 2:** Association and dissociation rates, dissociation constant K_D_: Gap peptides were injected over a surface with captured RadA-scaffolding protein, and the responses to dilution series were fitted to a 1:1 binding model. Fitted standard errors shown in parenthesis.

	k_a_ (1/ms)	k_d_ (1/s)	K_D_ (µM)
EL1-G27	10,000 (1000)	0.061 (0.004)	6
EL2-G26	511 (7)	0.0043 (0.0001)	8

## Data Availability

The data presented in this study are available on request from the corresponding author.
